# Reply to Lee, S.Y. Comment on “Krikorian et al. Early Intervention in Cognitive Aging with Strawberry Supplementation. *Nutrients* 2023, *15*, 4431”

**DOI:** 10.3390/nu16060825

**Published:** 2024-03-14

**Authors:** Robert Krikorian, Jeffrey Welge

**Affiliations:** Department of Psychiatry & Behavioral Neuroscience, University of Cincinnati Academic Health Center, Cincinnati, OH 45267, USA; jeffrey.welge@uc.edu

In his Comment [1] concerning our pilot clinical trial with strawberry supplementation in overweight, middle-aged individuals with subjective cognitive decline [2], Dr. Lee expressed the wish for more information and additional analyses to facilitate the implementation of the study approach in clinical practice. Dr. Lee detailed four specific concerns. The following discussion considers each of these issues.

It was noted that there was no analysis for gender differences. This was the case because of the small number of male participants and the unequal distribution of males between the groups. A total of 30 participants completed the trial and were included in the statistical analyses. All 15 participants randomized to the placebo group were female, and there were 10 females and 5 males randomized to the strawberry treatment group. Accordingly, there was no basis for evaluating the effect of between-group gender differences on the outcome measures. However, it is possible to compare the performances between the female and male participants on the pre-intervention baseline measures within the strawberry group. These analyses did not indicate sex differences with respect to the cognitive and mood measures, including executive ability (*p* = 0.57), lexical access (*p* = 0.94), verbal memory (*p* = 0.21), visual–spatial memory (*p* = 0.17), memory interference (*p* = 0.38), and depressive symptoms (*p* = 0.99). We also investigated the treatment responses of females relative to males within the strawberry group using ANCOVA (as performed in the original study). Aside from a strong trend (*p* = 0.06) indicating improved visual–spatial memory performance for male participants, we found no gender effect for the other outcomes; executive abilities (*p* = 0.34), lexical access (*p* = 0.37), verbal memory (*p* = 0.90), memory interference (*p* = 0.64), and depressive symptoms (*p* = 0.32).

Dr. Lee also raised concerns about the possibility of non-normal score distributions, in particular with regard to HOMA-IR (homeostasis model assessment of insulin resistance) values, a calculated index of insulin resistance [3]. The ANCOVA analyses are relatively unaffected by non-normality. However, in order to respond to Dr. Lee’s primary concern in this regard, we assessed the normality of the HOMA-IR distribution of scores at the enrollment and final study visits and found that the distributions at both assessment points were skewed (Figure 1). Given this, we log-transformed both score distributions and performed the ANCOVA to assess for changes in calculated insulin resistance with the intervention. However, as with the original analysis with non-transformed values, there was no effect of the strawberry intervention on HOMA-IR (*p* = 0.27).

Another concern raised by Dr. Lee was that performance differences in the outcome measures at baseline might not have been controlled or adjusted for in the statistical analyses. However, such differences were controlled in the ANCOVA applied to these outcome measures. These analyses involved between-group comparisons of the final visit cognitive and mood scores with covariate control for the corresponding baseline scores. In this way, we sought to isolate the effect of the intervention on cognitive performance and mood regardless of the level of baseline performance or differences between groups [4].

Finally, Dr. Lee noted an absence of discussion of side effects or adverse outcomes. There is very little to report in this regard. One participant in the strawberry-treated group reported constipation but managed to complete the final assessments. There was no other report of adverse effects. 

We hope this additional information will be helpful. Like most human berry trials, this study was a pilot trial. While such smaller trials are more susceptible to random effects and nil findings [5], they do contribute to the empirical knowledge base for understanding berry health benefits. 

## Figures and Tables

**Figure 1:**
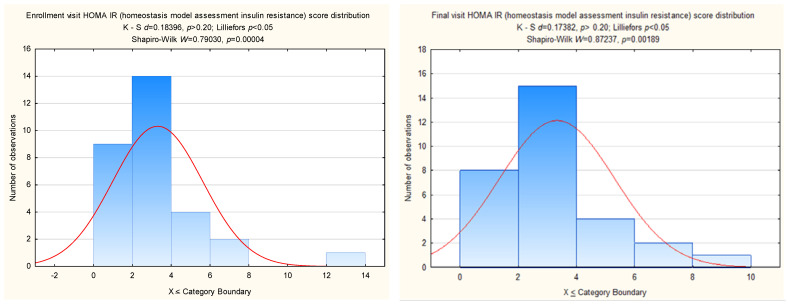
(The **left** panel) shows the distribution of HOMA-IR raw scores determined from samples obtained at the pre-intervention enrollment visit. (The **right** panel) shows the HOMA-IR raw score distribution determined from samples obtained at the final study visit. Both distributions are modestly skewed.
